# Intravital Two-Photon Microscopy of Immune Cell Dynamics in Corneal Lymphatic Vessels

**DOI:** 10.1371/journal.pone.0026253

**Published:** 2011-10-20

**Authors:** Philipp Steven, Felix Bock, Gereon Hüttmann, Claus Cursiefen

**Affiliations:** 1 Department of Ophthalmology, University of Lübeck, Lübeck, Germany; 2 Institute of Anatomy, University of Lübeck, Lübeck, Germany; 3 Department of Ophthalmology, University Clinic of Cologne, Cologne, Germany; 4 Schepens Eye Research Institute, Harvard Medical School, Boston, Massachusetts, United States of America; 5 Institute of Biomedical Optics, University of Lübeck, Lübeck, Germany; Massachusetts General Hospital and Harvard Medical School, United States of America

## Abstract

**Background:**

The role of lymphatic vessels in tissue and organ transplantation as well as in tumor growth and metastasis has drawn great attention in recent years.

**Methodology/Principal Findings:**

We now developed a novel method using non-invasive two-photon microscopy to simultaneously visualize and track specifically stained lymphatic vessels and autofluorescent adjacent tissues such as collagen fibrils, blood vessels and immune cells in the mouse model of corneal neovascularization in vivo. The mouse cornea serves as an ideal tissue for this technique due to its easy accessibility and its inducible and modifiable state of pathological hem- and lymphvascularization.

Neovascularization was induced by suture placement in corneas of Balb/C mice. Two weeks after treatment, lymphatic vessels were stained intravital by intrastromal injection of a fluorescently labeled LYVE-1 antibody and the corneas were evaluated in vivo by two-photon microscopy (TPM). Intravital TPM was performed at 710 nm and 826 nm excitation wavelengths to detect immunofluorescence and tissue autofluorescence using a custom made animal holder. Corneas were then harvested, fixed and analyzed by histology.

Time lapse imaging demonstrated the first in vivo evidence of immune cell migration into lymphatic vessels and luminal transport of individual cells. Cells immigrated within 1–5.5 min into the vessel lumen. Mean velocities of intrastromal corneal immune cells were around 9 µm/min and therefore comparable to those of T-cells and macrophages in other mucosal surfaces.

**Conclusions:**

To our knowledge we here demonstrate for the first time the intravital real-time transmigration of immune cells into lymphatic vessels. Overall this study demonstrates the valuable use of intravital autofluorescence two-photon microscopy in the model of suture-induced corneal vascularizations to study interactions of immune and subsequently tumor cells with lymphatic vessels under close as possible physiological conditions.

## Introduction

Lymphatic vessels are essential for maintaining the homeostasis of tissue-fluids, transport of antigen and migration of immune cells under physiological and pathological conditions. However, following organ or tissue transplantation, lymphangiogenesis triggers the rejection of transplanted organs or tissues and thereby limits transplant survival [1], [2]. Furthermore, the formation of lymphatic vessels during tumor growth increases the risk of tumor metastasis to adjacent lymph nodes and beyond [3]. The precise molecular and cellular interactions governing these important cell-vessel interactions are only poorly understood until now.

Lymphangiogenesis research lacked behind hemangiogenesis research for several decades and only relied on electron microscopy due to the absence of specific markers for tissue staining. Since specific markers for lymphatic vascular endothelium such as LYVE-1, Podoplanin and Prox1 were introduced in the late 1990s, lymphangiogenesis research has made great progress and now includes ex vivo fluorescence and confocal microscopy on tissue sections and in-vitro assays (tube forming [4], transwell [5] or proliferation assays [6]) to investigate the structure of lymphatic vessels and the interaction with their environment. Nevertheless cellular dynamics such as migration of immune cells or tumor cells into lymphatic vessels and further migration within the vessels cannot be investigated in fixed tissue. Recently Pflicke and Sixt demonstrated for the first time, that isolated DCs migrate through preformed gates into lymphatic vessels in an in situ murine ear sheet model [7]. However, such ex vivo models or organ cultures have particular limitations in terms of perfusion and innervation and the in vivo situation might differ significantly. Therefore high-resolution intravital imaging techniques are desirable for the detection and analysis of cell-cell and cell-vessel dynamics under conditions as close to physiology as possible.

The cornea of the eye is a physiologically transparent and avascular tissue [8], consisting out of densely packed collagen fibrils with almost no scattering properties. This tissue is perfectly suitable for microscopic investigations and also easily accessible in the living animal. Within the physiologically avascular cornea hem- and lymphangiogenesis can be stimulated using the model of suture induced corneal inflammation. Through this, invading blood and lymphatic vessels are applicable for experimental analysis and manipulation under controlled conditions [1] [6] [9], [10], [11]. The transparent cornea further allows to image immune cells such as corneal dendritic cells (DCs) [12], [13] or intravascular leucocytes at the corneal limbus or the iris [13]. These studies however focused on cell-blood vessel interaction or migration of DCs rather than cell-cell or cell-lymphoid vessel interaction and required labeling of cells and or use of intravascular dextran injection. In these studies the use of epifluorescence in vivo microscopy or confocal microscopy also limits the observer's ability for spatial orientation and depth determination or relies on the subsequent ex vivo detection of the structures [14].

In 1990 Denk et al. introduced two-photon microscopy for intravital high-resolution imaging in large tissue depths [15]. Since then numerous studies have investigated cell-cell interactions in lymph nodes and spleen by intravital two-photon microscopy. Using two-photon microscopy Yaniv et al. were also able to demonstrated lymphatic vessels in a transgenic zebrafish [16]. As data on mammals are not available up to this point, a model for specifically and simultaneously imaging lymphatic vessels and all key-players in an immune reaction (immune cells, blood vessels, connective tissue) is highly desirable.

The aims of this study were to develop an experimental setup that enables detecting lymphatic vessels, blood vessels, immune cells and surrounding tissue components simultaneously and to analyze cell-lymphatic vessel interactions in vivo. In particular, we hypothesize that in a living animal immune cells within the inflamed cornea demonstrate active migration patterns and use openings of lymphatic vessels to emigrate from the cornea.

## Results

### Imaging the lymphatic vessel network within the corneal microenvironment

**Figure 1:**
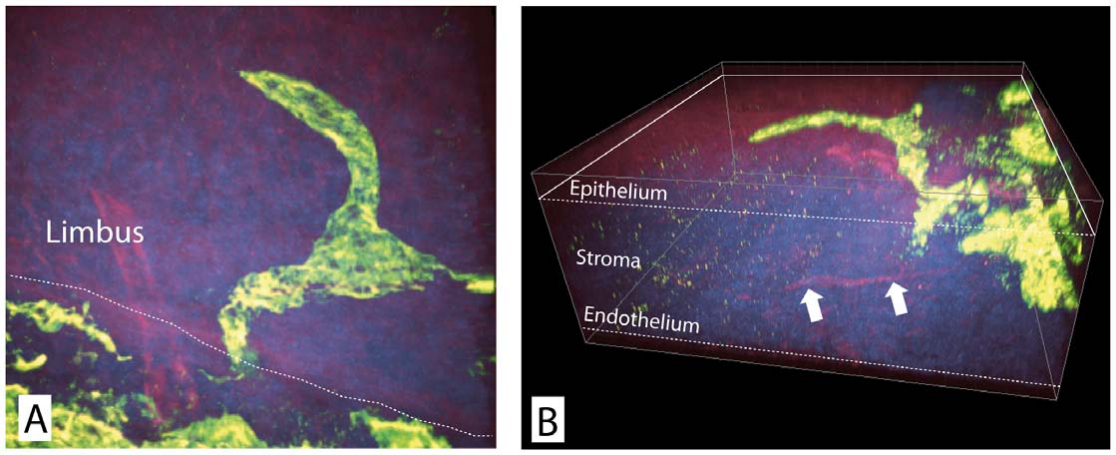
3D reconstruction of the lymphatic arcades ex vivo. A) At the limbus, the junction between the epithelium of conjunctiva and cornea (dotted line), lymphatic vessels (green) invade into the corneal stroma (blue) and are primarily located directly below the basement membrane extending towards the central cornea (B). Nerve fibers stretching into the stroma are also visible (B, arrows).

Initially we used two-photon microscopy to analyze the morphology of corneal lymphatic vessels and their vicinity ex vivo. Therefore we induced the ingrowth of pathological blood and lymphatic vessels by placing sutures intrastromally into the corneas of BALB/c mice. Ex vivo two-photon microscopy enabled a simultaneous detection of tissue autofluorescence and ALEXA-488 LYVE-1 stained lymphatic vessels and allowed to depict the entire corneal microenvironment consisting of epithelium, corneal stroma with collagen fibrils, nerve fibers, lymphatic vessels and blood vessels. Recording image stacks through ex vivo prepared whole mounted corneas with 1 µm steps demonstrated invading lymphatic vessels that sprouted at the limbus (junction between the epithelium of conjunctiva and cornea) into the corneal stroma and that were primarily located below the basement membrane while extending towards the central cornea (Fig. 1, Video S1).

### Design and development of intravital two-photon setup

**Figure 2:**
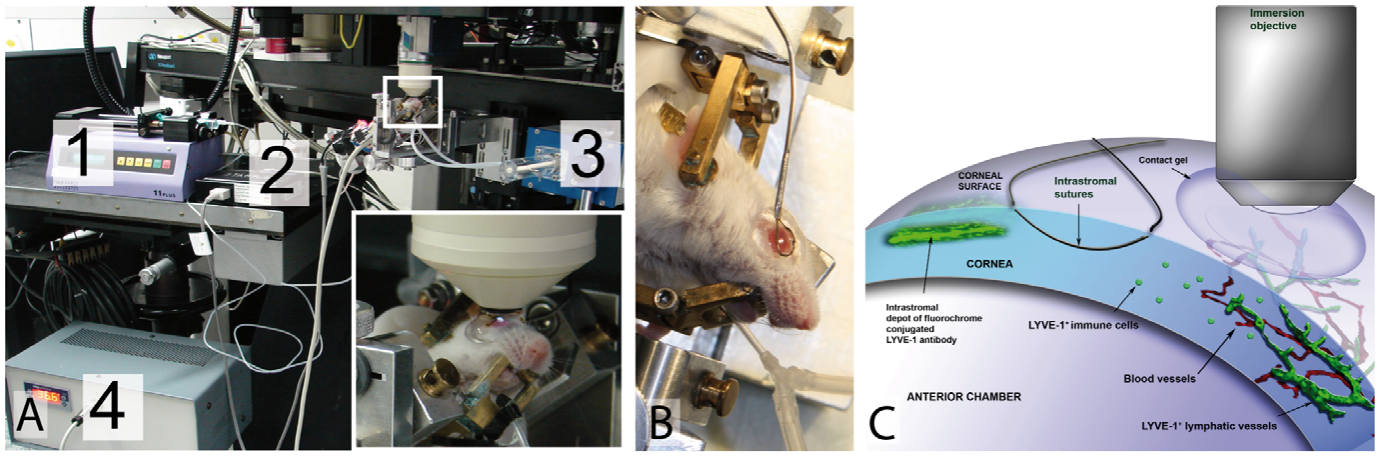
Experimental setup for intravital 2-photon microscopy. A) 1: intraperitoneal anesthesia/infusion pump; 2: monitoring blood oxygen, pulse and breath rate (by MouseOx; 3: ventilation by tracheotomy followed by intubation; 4: body temperature control; Inset: custom made animal holder. B) Reduction of artificial pulsatile movements by a custom made eye globe clamp. C) Scheme for intravital visualization of specifically stained lymphatic vessels and autofluorescent blood vessel and tissue in the murine cornea. Intrastromally injected LYVE-1/Alexa 488 antibody binds specifically to suture induced lymphatic vessels. For non-contact intravital observation a 20× immersion objective dipped into artificial tear gel is used.

As cellular velocities vary under changing conditions such as temperature [17] and oxygen saturation (own unpublished findings) our setup was designed to control these factors using a custom made animal holder equipped with a heating mat and by monitoring blood oxygen levels, pulse and breath rate (Fig. 2A). Artifacts during intravital observations based on pulse synchronal movement of the eye globe were minimized by deep anesthesia and immobilization of the cornea by a specific clamp (Fig. 2B). Remaining slight drift artifacts were compensated by semiautomated alignment using the piezodriven objective holder and the motor driven object table and by limiting recording times to 15 minutes. Together with a software based realignment of the image stacks or series high resolution data sets were generated. The setup used enabled to anesthetize an individual mouse for up to 5 hours and perform several intravital examinations including image stacks or videos to screen the entire cornea and detect rapid cellular movements. Repeated recording of the same area of interest lead to very limited photobleaching and by using excitation energies below 40 mW no phototoxic effects occurred. Nevertheless, if photobleaching effects emerged the ALEXA 488 fluorophore bleached faster than the intrinsic tissue autofluorescence of e.g. epithelial cells. Signals from sutures and stromal collagen remained stable even if excitation energies were tuned to 80 mW and above.

### Intravital specific visualization of lymphatic vessels

**Figure 3:**
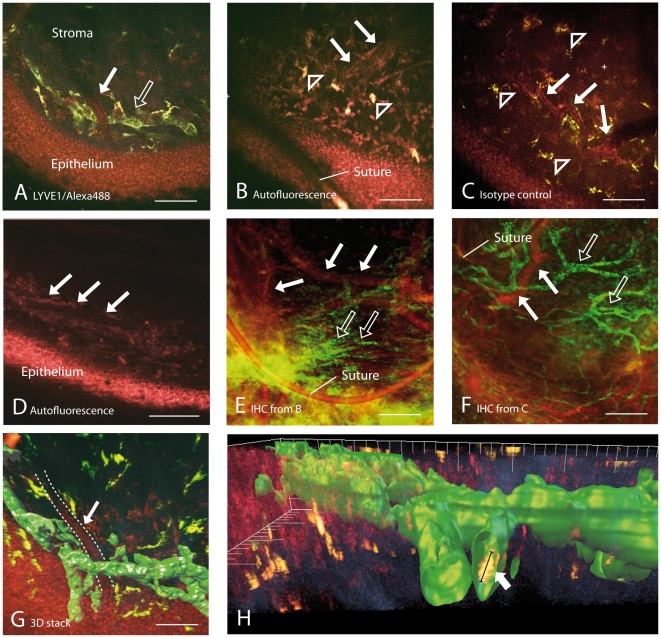
Intravital visualization of lymphatic vessels in the subepithelial stroma of the cornea. A) Intravital image of a lymphatic vessel in an experimentally vascularized murine cornea. The fluorochrome labeled lymphatic vessel (open arrow) is shown in green and autofluorescence of epithelium, blood vessel (white arrow) and tissue cells is shown in red, surrounding collagen fibrils in blue. B) Intravital image of unstained vascularized cornea. Tissue autofluorescence reveals individual cells (red) and blood vessels (red, white arrows). Some cells feature autofluorescence in all detection channels (yellow-white, arrowheads). Lymphatic vessels cannot be visualized by autofluorescence excitation. C) Intravital image of isotype control injected cornea. Individual cells (red) and blood vessels (white arrows) are visible. Large cells feature green fluorescence deriving from intracellular Alexa488 fluorochrome. Lymphatic vessels are not labeled by isotype controls. D) Intravital autofluorescence image of normal cornea at the limbus. A limbal blood vessel is visible (white arrows) and few individual cells (red). E) Ex vivo confocal image of specimen B) following immunohistochemistry (IHC). CD31+ blood vessels (red; white arrows) and Lyve-1+/CD31− lymphatic vessels (green, open arrows) are visible. F) Ex vivo confocal image of specimen C) following IHC. CD31+ blood vessels (red; white arrows) and Lyve-1+/CD31− lymphatic vessels (green, open arrows) are now visible. G) 3D-reconstruction of a 2-photon image stack through the vascularized cornea. The signal of the fluorochrome labeled lymphatic vessels is virtually reconstructed and depicted by surface rendering (green). Surrounding collagen fibrils are displayed in blue, epithelial cells and the blood vessel (dashed line and arrow) in red. H) Higher magnification and different angle of G). Within the lymphatic vessel an Alexa488^+^ presumed macrophage (arrow) is depicted in yellow as an overlay of Alexa488 fluorophore (green) and autofluorescence signal (red, diameter: 16.7 µm). Images recorded with acquisition times of 13.4s/image.

In vivo injection of ALEXA-488 conjugated LYVE-1 antibodies into a stromal pocket of a previously vascularized cornea was followed by drainage of the dye by the ingrown lymphatic vessels and specific adherence of the green-fluorescing antibodies to the lymphatic endothelial cells (Fig. 2C). The green-fluorescing antibody stained lymphatic vessels up to their finest protrusions and enabled a three dimensional reconstruction of the vessel lumen. As shown in Figure 3 lymphatic vessels were not detectable in non-injected mice (Fig. 3B), isotype-control injected mice (Fig. 3C) and limbal regions of normal non-sutured mice (Fig. 3D) in vivo. However, follow-up ex vivo immunohistochemistry of non-injected and isotype-control injected corneae that were previously sutured (Fig. 3B and C) revealed the existence of lymphatic vessels in the pre-examined areas of these specimens (Fig. 3E and F).

Corneal sutures induce an upregulation of proinflammatory chemokines with consecutive influx of inflammatory lymphocytes, corneal edema and increased conjunctival blood flow [18]. Also, corneal sutures resemble the preferential location for T-cells and macrophages for up to 4 weeks post surgery [19]. Therefore, areas in close proximity to the sutures were chosen for real-time imaging in our experiments. Sutures excited a specific strong autofluorescence signal that enabled an easy detection as landmarks within the area of interest (Fig. 3 B, E, F and Video S1).

### Intravital detection of immune cell migration in relation to lymphatic vessels

2D time series were generated by repeatedly recording image sections of the same tissue plain. Within this pilot study recording times of up to 15 minutes/series were chosen.

2D time series demonstrated extensive cellular migration within the corneal stroma close to but also far from lymphatic and blood vessels. The rapid movement of intravascular erythrocytes prevented to identify single cells and resulted in a specific motion artifact (continuous band of fluorescence with darker stripes) that furthermore documented intact perfusion during the experiments (Fig. 3A, Video S2 and S3). Migrating cells within the extracellular matrix (ECM) demonstrated a strong cytoplasmic autofluorescence signal that could be detected by the second and the third channel (excitation 450–500 nm, 500–580 nm) in our setup. These cells featured cellular sizes of 10–15 µm and an amoeboid migration pattern with average velocities of 9.5 µm/min (max. speed: 54 µm/min; Tab. 1). Numerous cells obviously followed preformed paths through the corneal stroma and interacted with other cells (Video S2). In addition cells with diameters of up to 30 µm featuring dendrites that protruded far into the periphery were present. These cells showed no migration over the periods recorded but were constantly contacted by mobile cells (Video S2).

**Table 1:** Migration velocities of intrastromal immune cells.

Migration (n = 50)	
Speed max. (µm/min):	54.0
Speed min. (µm/min):	0
Speed avg. (µm/min):	9.5

In each recorded time series a number of immune cells was traced and their average migration speed was calculated (*number of migrating cells: 50; speed max: maximal distance a single cell migrated within a minute in the corresponding time series; speed avg: average of all migration velocities measured in this time series*).

### Intravital visualization of cell migration across lymphatic vessel walls

**Figure 4:**
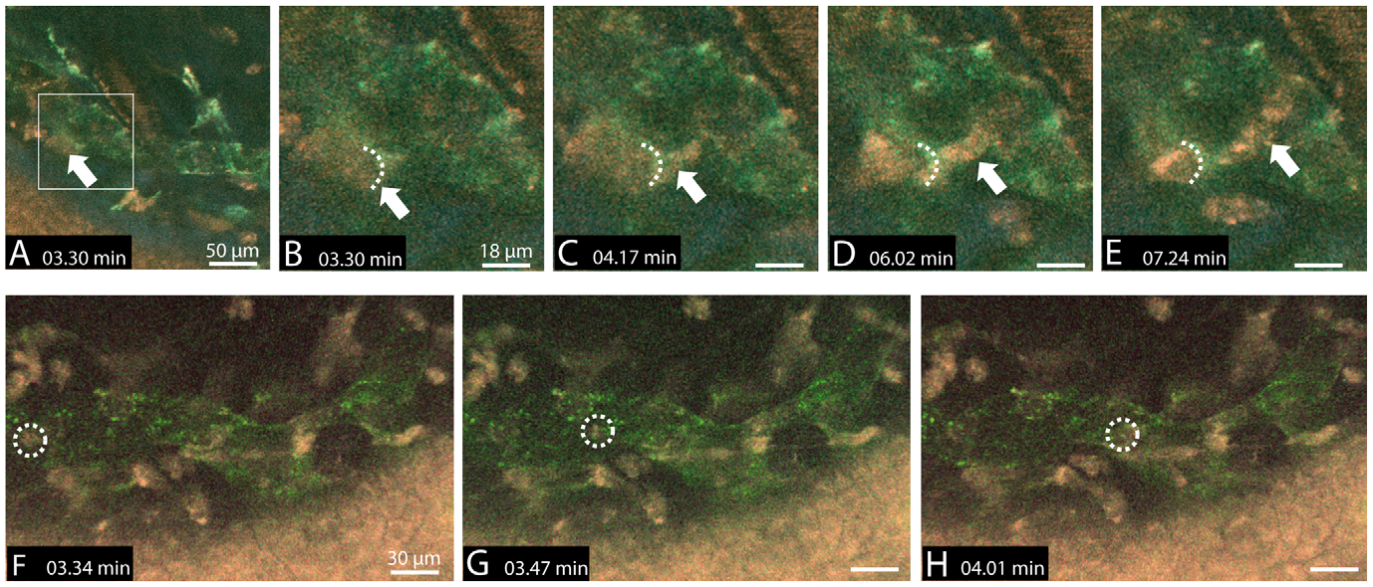
Intravital visualization of immune cell migration into and within a lymphatic vessel in a pathologically vascularized cornea. A–E) Lymphatic vessel (green), crossing blood vessel (red), corneal epithelium (red) and stromal collagen fibrils (blue) are imaged simultaneously. An individual cell (arrow) migrates into the lymphatic vessel via a presumed gate (dotted line) that demonstrates an enhanced LYVE-1-antibody signal (compare to video S3). F–H) Rapid intravascular transport of a single cell (dotted circle).

Besides ECM patrolling cells we were also able to observe the transmigration of highly motile single cells migrating into LYVE-1 labeled lymphatic vessels through presumed gates that demonstrated an enhanced fluorochrome signal (Fig. 4A–E, Video S3, Tab. 2).

**Table 2:** Transmigration velocities of immune cells into lymphatics.

Transmigration (n = 7)	
Speed max. (µm/min):	41.6
Speed min. (µm/min):	0
Speed avg. (µm/min):	11.4
Speed avg. before transmigration (µm/min):	8.9
Speed avg. after transmigration (µm/min):	10.4

Cells that migrated through the vessel wall into the lumen of the lymphatic vessel were traced in three separate time series and migration speed before, during and after transmigration was analyzed. (*number of transmigrating cells: 7; speed max: maximal distance a single cell migrated within a minute in the corresponding time series; speed avg: average of all migration velocities measured in this time series*).

**Figure 5:**
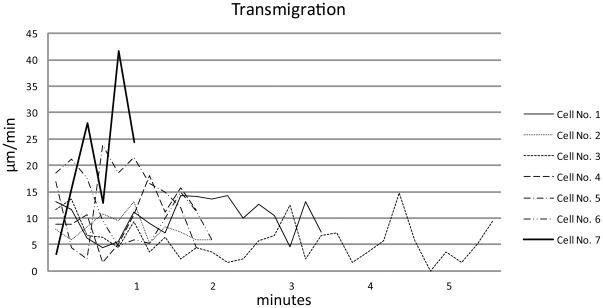
Velocity characteristics of the transmigrating cells. Cell tracking from first contact of leading cell protrusion with the lymphatic vessel until last contact with rear protrusion after passage. Cells 1–6 demonstrate velocities with min/max of 0–23 µm/min. One cell (no. 7) shows rapid migration velocities with a maximum of 41.6 µm/min. This cell was the only cell transmigrating from the lymphoid vessel into the stroma. Transmigration process required 1–5.5 min.

**Figure 6:**
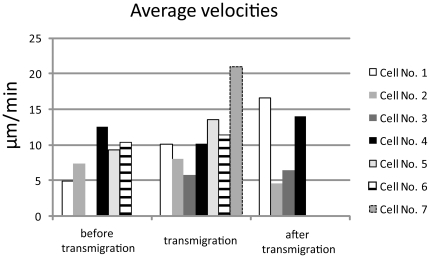
Average velocities of transmigrating cells correlated to transmigration process. Classification: before transmigration: track until first contact of leading protrusion with the lymphatic vessel wall, transmigration: track from first until last contact of migrating cell with the lymphatic vessel wall, after transmigration: track from last contact of migrating cell with lymphatic vessel wall. No values could be obtained for cells no. 3+7 before transmigration and cells 5–7 after transmigration, due to transmigration-limited recording periods.

These cells featured a leading protrusion that entered the lymphatic vessel and the rest of the cell then squeezed through the gate, hereby following closely the path of the preceding cell. The entire process of transmural migration took 1–5.5 minutes at average velocities of 11.4 µm/min (Fig. 5). Velocities before transmigration averaged 8.9 µm/s and 10.4 µm/min after transmigration (Tab. 2 and Fig. 6). These cellular transmigration activities demonstrated the first unequivocal in vivo evidence of corneal lymphatic vessel function to facilitate transmural immune cell migration. In addition, some cells located closely to the gate did not enter the vessel, indicating a selective entry mechanism of individual cells., Following transmigration into the lymphatic vessel immigrated cells remained intravascular for periods of 3–7 minutes (overall video duration 12–16 minutes) and no afferent transport to the periphery was observed following the entry of the cell. These intravascular cells demonstrated a diameter of approximately 16 µm and featured both a fluorochrome- and an autofluorescence-derived signal (Fig. 3E) implicating that these cells either accumulated the injected antibody within their cytoplasm or were specifically labeled with the LYVE-1 antibody. These assumed macrophages were also detected throughout the corneal stroma with focal accumulations close to the sutures (not shown). In contrast to transmigrating cells, other individual cells within the lymphatic vessels demonstrated rapid dislocations with maximum velocities of 224 µm/min (Fig. 4F–H, Tab. 3).

**Table 3:** Intravascular transport of immune cells.

Intravascular transport (n = 3)	
Speed max (µm/min):	224.4
Speed min. (µm/min):	0
Speed avg. (µm/min):	101.5

Intravascular cells were traced and velocities were calculated in two separate time series. (*number of transported cells: 3; speed max: maximal distance a single cell was transported within a minute in the corresponding time series; speed avg: average of all transportation velocities measured in this time series*).

### Presence of immune cells in the vascularized cornea

**Figure 7:**
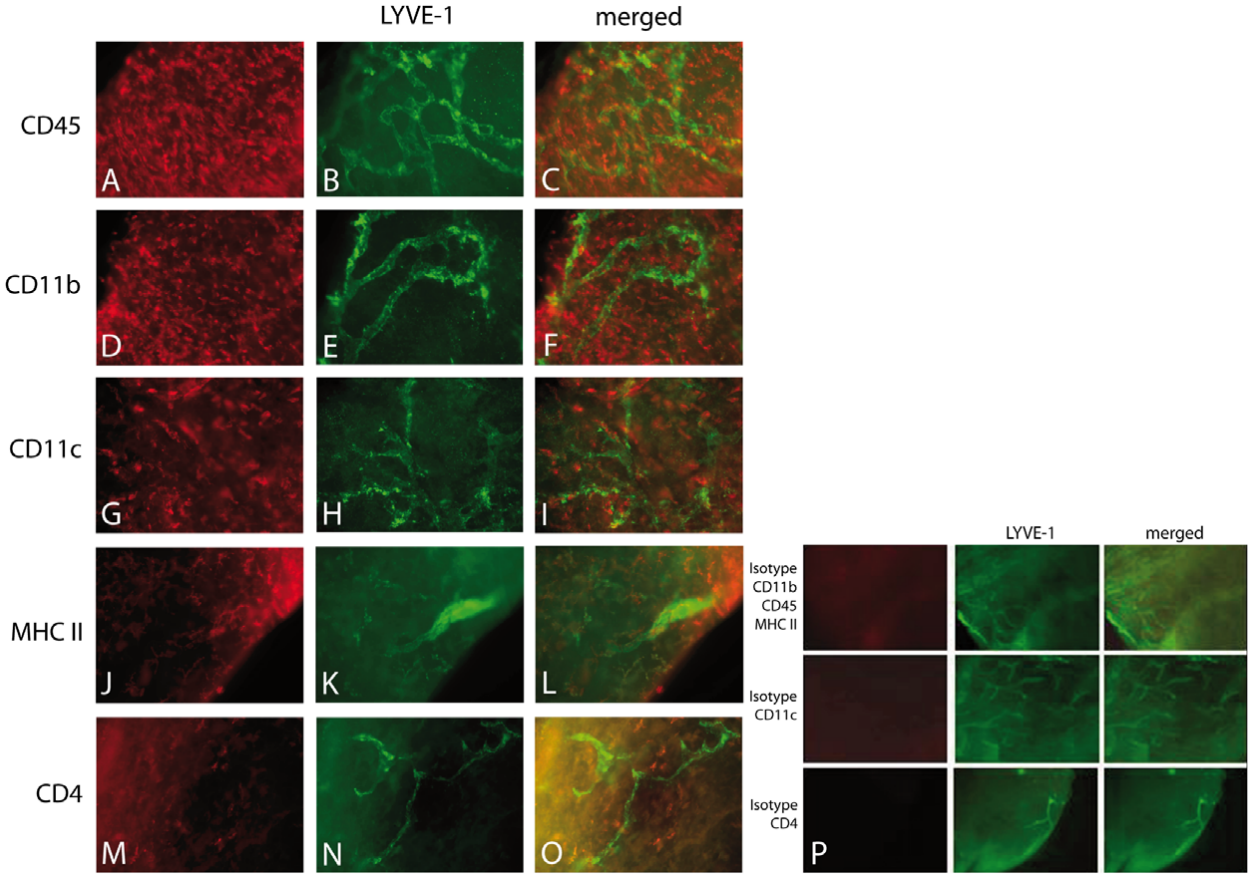
Presence of antigen presenting cells and lymphocytes near corneal lymphatic vessels. Numerous leucocytes are located within the vascularized cornea (A–C: CD45), consisting of CD11b+ macrophages (D–F), CD11c+ dendritic cells (G–I), MHCII+ antigen-presenting cells (J–L) and CD4+ T-cells (M–O); Isotype controls (P), *Magnification 200×.*

To further characterize migrating and resting cells in this model vascularized murine corneae were stained using a panel of different antibodies (Fig. 7). The corneas revealed a dense accumulation of CD45+ leucocytes in close proximity to the lymphatic vessels. These cells were mainly CD11b+ [11] macrophages and to a lesser extend CD11c+ dendritic cells in concordance to earlier studies of the inflamed cornea [20], [21]. Few MHCII+ antigen-presenting cells (APCs) were present and featured a dendritic shape. CD4+ T-cells were also located in close relation to the lymphatics, but limited in number.

## Discussion

Lymphatic vessels play a pivotal role in physiological and pathological conditions such as tissue-fluid homeostasis, immunological defense of infections, transplant rejection and tumor cell migration. To understand the function of resident or newly formed lymphatic vessels besides molecular mechanisms, cell-cell and cell-vessel interactions need to be analyzed in detail. Hereby, imaging lymphatic vessels in vivo together with blood vessels and the surrounding microcompartment is a mandatory requirement to analyze any dynamic interaction e.g. during inflammation or tumor growth. In contrast to the facile detection of blood vessels, the main limitation of visualizing lymphatics in vivo is the lack of contrast due to morphological characteristics of the vessels. Other than blood vessels that consist of three layers (intima, media, adventitia), lymphatic capillaries consist of a single cell layer (endothelium), which is anchored by collagen fibrils in the extracellular matrix. In confocal laser scanning microscopy the delicate walls of the vessels excite only very weak autofluorescence and give only little contrast that is additionally outshined by surrounding collagen fibrils. In addition, the transparent lymph fluid does not contribute to the detection of these vessels. By combining injected fluorochrome-labeled antibodies into a stromal pocket with the detection of two-photon excited tissue autofluorescence, previously undetectable lymphatic vessels and adjacent tissue specific structures as well as individual cells became visible in vivo. In contrast to previously used fluorescing dextrans, this approach features long term labeling of the lymphatic vessel morphology in vivo, even after post-experimental tissue preparation [22]. It also allows analyzing individual cells within the optical clear lumen of the vessels. In addition, injection of anti-LYVE1 antibody into a stromal pocket is not limited to a particular mouse strain but can be applied to any transgenic mouse model established for immunological research.

Our setup enabled studying cellular dynamics within the model of suture induced neovascularization of the cornea repeatedly over several hours. Observations included migration of immune cells along restricted paths, which may be facilitated by preformed tunnels [23], [24] or partly along protrusions of resident dendritic cells. Cellular velocities of stromal immune cells measured in our experiments (9.5 µm/min at avg.) are comparable to T-cell velocities measured in isolated non-inflamed lymphnodes [17], T-cell velocities within an infiltrating tumor model [25] or macrophage velocities in a wound healing model of medaka fish [26]. As immunohistochemistry demonstrated only few T-cells in our model that were far outnumbered by macrophages, migrating cells are likely to be macrophages. As autofluorescence emitted by these cells is based on intrinsic fluorophores such as NAD(P)H and macrophages and also dendritic cells contain lysosomes that contribute to the autofluorescence signal, we selectively detected the different autofluorescence spectra of NAD(P)H and lysosomes in different channels in our setup. By analyzing such autofluorescence spectra and intensity differences, we were able to optical characterize cellular and also non-cellular structures as published previously e.g. for erythrocytes, connective tissue, macrophages [27] and stem cells [28].

The finding of normal cellular velocities in this study affirms the clinical appearance of corneae with only little edema and normal conjunctival blood flow at the time points used as inflammation was shown to increase velocities of immune cells significantly [25]. Besides increasing cellular velocities inflammation also increases cellular migration from the inflamed tissue via lymphoid vessels by upregulating leukocyte adhesion factors such as ICAM-1, VCAM-1 and E-selectin [5]. By this, an increased cellular turnover within the inflamed tissue is facilitated and accompanied by transmigration of immune cells via lymphatic vessel walls. In situ real-time dynamics of transmural migration of dendritic cells (DCs) into lymphatic vessels have recently been recorded by Pflicke and Sixt for the first time. Within their ex vivo ear sheet model, injected isolated DCs migrated into lymphatics by preformed pores [7]. However, in our experiments we observed the immigration of immune cells into lymphoid vessels for the first time in vivo (Fig. 4A–E, Video S3) and also recorded and analyzed the transport of immune cells within lymphatic vessels (Fig. 4F–H). As these intravascular dynamics are extremely unlikely to be based on active cellular migration, the data implicates a passive transport via the lymph flow. Only limited data is available on lymph flow velocities in mice. Measurements range from 84 µm–81 mm/min, partly much faster than the velocities measured in our experiments [29], [30]. Nevertheless, the data available is based on studies of the lymphatic vessel system of tail and limb. To our knowledge no studies on lymph flow velocities in the vascularized cornea of mice have been conducted so far and velocities in the corneal stroma that consists of densely packed collagen fibrils might differ significantly from lymph flow velocities in connective tissues of other regions.

Together with previous findings that lymphoid vessels in the model of suture induced corneal inflammation increase the risk of immunological transplant rejection following corneal transplantation, the demonstrated migration and intravascular transport of immune cells and the continuous staining pattern proves the functionality of these newly formed lymphatic vessels and their ability of draining foreign matter such as injected dyes or even antigen.

Based on the setup and the data presented future experiments are planned to target two important issues: 1. How do immunosuppression and anti-angiogenesis influence cellular and vascular dynamics in connection to the level of inflammation? 2. The development of a setup that allows detecting and analyzing lymphatics and cellular dynamics without the necessity of manipulation such as dye injection as a requirement for studies in humans.

Overall, this method paves the way for new intravital analyses of interactions of the immune system and lymphatic vessels as well as tumor cells and lymphatic vessels which has been of major scientific relevance in the last years [31].

## Materials and Methods

### Mice and anesthesia

Six to eight weeks-old female Balb/C mice (Charles River Germany, Sulzfeld, Germany) were used. All animals were treated in accordance with the ARVO Statement for the Use of Animals in Ophthalmic and Vision Research. For surgical procedures, mice were anaesthetized using an intraperitoneal injection of Ketamine (Ketanest S, Parke-Davis, Germany) and Xylazine (Rompun, Bayer, Germany).

### Suture induced corneal inflammation assay

We used the mouse model of suture induced inflammatory corneal neovascularization as described previously [6]. Briefly, mice were put under general anesthesia and 3 intrastromal 11-0 nylon sutures (70 µm diameter needles; Serag-Wiesner, Naila, Germany) were placed in the corneal stroma with two incursions extending over 120° of corneal circumference each. After two weeks corneal neovascularizations extended towards the sutures and animals were investigated by means of ex vivo and intravital two-photon microscopy.

### Corneal immune cell staining

14 days after suture placement, corneas were harvested and fixed in acetone for 15 min at 4°C. After three washing steps the specimens were blocked with 2% BSA in PBS for 15 min and afterwards with FC-Block (CD12/CD36; Invitrogen, USA) for 30 min at 4°C.

The specimens where stained with the primary antibodies over night at 4°C. We used LYVE-1 (rb α ms, AngioBio, USA; preconjugated with FITC or with Cy3 goat α rabbit, Dianova, Germany) for lymphatic vessels, CD4 (rb α ms, Santa Cruz, USA) for T-cells, CD11b (FITC rt α ms, Serotec, USA) for tissue macrophages, CD11c (FITC hamster α ms, AbD serotec, UK) for dendritic cells, MHC II (PE rat α ms I-A/I-E; BD Pharmingen, USA) for antigen presenting cells and CD45 (FITC rat α ms, BD Pharmingen, USA) as leukocyte marker. According isotype controls: normal-rat (Santa Cruz, USA); normal-hamster (Santa Cruz, USA); normal-rabbit (Abcam, USA). At day two the secondary antibody was applied for 45 min at room temperature.

### Intravital two-photon microscopy

We used a commercially available, modified two-photon microscope (DermaInspect JenLab GmbH, Neuengönna, Germany). The DermaInspect consisted of a solid-state, mode-locked 80 MHz Ti:sapphire laser (MaiTai, Spectra Physics, Darmstadt, Germany) with a tuning range of 710–920 nm and a mean laser output of >900 mW at 800 nm which delivered pulses with a width of approximately 150 fs to the sample. Setting of the excitation power and beam steering were done by a computer-controlled beam attenuator, a shutter, and a two axis galvoscanner. For in-vivo imaging of comparatively large image volumes with a high resolution a 20× objective (Plan-Apochromat DIC 20/1.0 W objective, Carl Zeiss GmbH, Jena, Germany), which was focused by a piezodriven holder, was chosen. Larger scale motions of the sample in x- and y-directions were performed by computer-controlled stepper-motors (Owis GmbH, Staufen, Germany). After passing through a specially designed main beam splitter with high transmission between 350 nm und 710 nm (Layertec, Mellingen, Germany) the fluorescence was detected in parallel in four spectral ranges (380–450 nm, 450–500 nm, 500–580 nm, 580–680 nm) by a combination of dichroic beam splitters (ST440CCXR, HCBS495 und ST580DCXR, AHF analysentechnik AG, Tübingen), a blocking filter (E680SP, AHF analysentechnik AG, Tübingen) and four photomultipliers (R1924 and R1925, Hamamatsu, Herrsching, Germany). Three of the four spectral ranges (channel 1–3) were used in these experiments. Channel 1 mainly detected autofluorescence and second-harmonic generation (SHG) signals from collagen, channel 2 detected autofluorescence signals from cells and structures except collagen and channel 3 detected the fluorescing antibody.

Prior to examination, a LYVE-1 antibody (AngioBio Co., Del Mar, CA, USA) or the according isotype control (Rabbit IgG, Abcam, USA) was conjugated with the fluorochrome Alexa 488 (Alexa Fluor 488 Monoclonal Antibody Labeling Kit, Invitrogen, Paisley, UK) and a volume of 5 µl was injected into a stromal pocket in the prevascularized murine cornea. Intravital examination was conducted 24 hours after intrastromal antibody injection.

### Custom made animal holder

For the intravital investigations, a custom made animal holder was used to secure and orientate the mice (Fig. 2A+B). The animal holder was equipped with a heating device to maintain normal body temperature and additional monitoring of the blood oxygen level, pulse and breath rate was facilitated by a murine pulse oximeter system (MouseOx, Starr Life Sciences Corp., Pittsburgh, PA, USA). The animals were anesthetized by constantly infusing 0.2–0.3 ml/h of a solution of 300 µl fentanyl, 400 µl midazolame, 200 µl domitor and 3.5 ml sodiumchloride via an intraperitoneal catheter. Controlled ventilation was conducted via tracheotomy followed by intubation at settings of 180–250 strokes/min and 180–220 µl/stroke (MouseVent, Harvard Apparatus, Hugo Sachs Elektronik, Germany), maintaining SO_2_ levels of 90–98%.

The secured, heated, ventilated and monitored mouse was placed beneath the two-photon microscope and the eye was covered with an artificial tears gel (Vidisic Gel, Bausch &Lomb/Dr. Mann Pharma, Berlin, Germany) to bridge the working distance of the water immersion objective used.

### Image analysis

Image stacks of the vascularized cornea were made at 730 nm excitation wavelength and comprised up to 80 images and volumes up to 200×200×70 µm. To obtain information about the cellular dynamics in living animals, time series were made in one image plain, consisting of up to 80 images (one image every 4.4, 11.7 or 13.4 seconds). Image stacks and series were analyzed using Imaris software 6.5 (Bitplane, Switzerland). Datasets of three spectral ranges were used for analysis and color coded as follows: 380–450 nm: blue, 450–500 nm: red, 500–580 nm: green. For statistical analysis of cellular dynamics, individual cells were tracked manually using Imaris and tracks were generated automatically. Minimum speed in each time series was 0, as at least one cell showed a rest of motility at a given timepoint, e.g. during cell-cell interaction. Maximum speed resembles the maximum dislocation of a single cell in one time series.

## Supporting Information

Video S1 **3D-reconstruction of an image stack displaying intravital imaging of lymphatic vessels and immune cells in the cornea, recorded by 2-photon microscopy.** Intrastromal sutures (bright red) induce lymphatic vessels (green) within the superficial stroma of the cornea, below the corneal epithelium (red). By injecting an Alexa 488-conjugated anti-LYVE-1-antibody into an intrastromal pocket, the draining lymphatics are labeled by the antibody and imaged by 2-photon microscopy to the point of their finest branches. Blue: collagen fibrils (grid spacing 10 µm).
(MOV)

Video S2 **Time series on corneal lymphatic vessels induced by intrastromal sutures detected by intravital 2-photon microscopy.** Autofluorescent immune cells (red) migrate rapidly within the corneal stroma and around anti-LYVE-1-antibody labeled lymphatic vessels (green) and stationary cells with long dendrites (arrows). Some cells (red spheres) follow presumed preformed paths (grey lines). (Time series 15 min 11 s, acquisition time 13.4s/image).
(MOV)

Video S3 **Intravital 2-photon microscopy of suture-induced lymphatic vessels within the cornea.** By using a 2-photon microscope, equipped with a four channel detector, epithelial cells, stromal collagen, individual immune cells, blood vessels and fluorochrome-conjugated antibody labeled lymphatic vessels can be investigated simultaneously. In this time series an individual cell (arrow) migrates into the lymphatic vessel via an opening in the vessel wall that is depicted by enhanced antibody labeling and consecutive enhanced fluorescence signal. (Time series: 16 min 46 s, acquisition time 11.7s/image).
(MOV)
